# P-2346. Mpox-Specific Cellular and Humoral immunity in Mpox-Survivors Living With HIV

**DOI:** 10.1093/ofid/ofae631.2498

**Published:** 2025-01-29

**Authors:** Samuel D Stampfer, Shainy Sambyal, Sailaja Gangadhara, Colleen F Kelley, Rama R Amara, Anandi N Sheth

**Affiliations:** Emory University School of Medicine, Atlanta, Georgia; Emory University School of Medicine, Atlanta, Georgia; Emory University School of Medicine, Atlanta, Georgia; Emory University, Decatur, GA; Emory University School of Medicine, Atlanta, Georgia; Emory University School of Medicine, Atlanta, Georgia

## Abstract

**Background:**

Mpox is an orthopoxvirus with a rodent reservoir that causes a disease similar to mild smallpox. In 2022, a sexually-transmitted global mpox outbreak infected over 90,000 individuals. Some immunocompromised patients experienced prolonged, severe mpox resulting in significant morbidity, amputations, and death. People living with HIV (PLWH) were at particularly high risk due to impaired cell-mediated immunity, as CD4 and CD8 T-cells help eradicate the virus from infected lesions.

**Figure:**
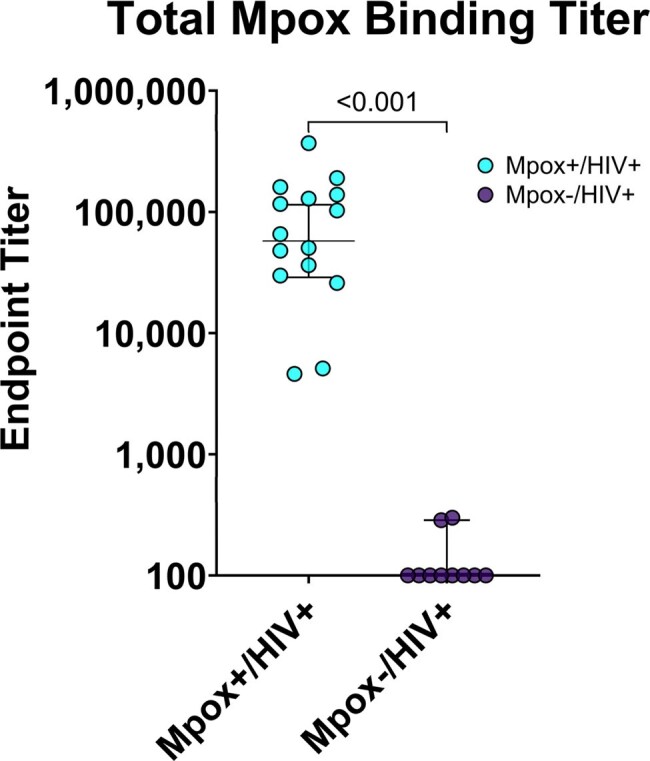

Mpox virion total binding titer in PLWH with or without history of mpox infection

**Methods:**

We compared immune responses to mpox in PLWH who were either mpox-naïve (n = 10) versus mpox-survivors (n = 15). We measured total mpox antibody levels with a novel ELISA assay using lysed mpox virions. We characterized mpox and orthopoxvirus-specific cell-mediated immunity using intracellular cytokine staining with an enhanced protocol to allow detection of low-frequency cellular populations.

**Figure:**
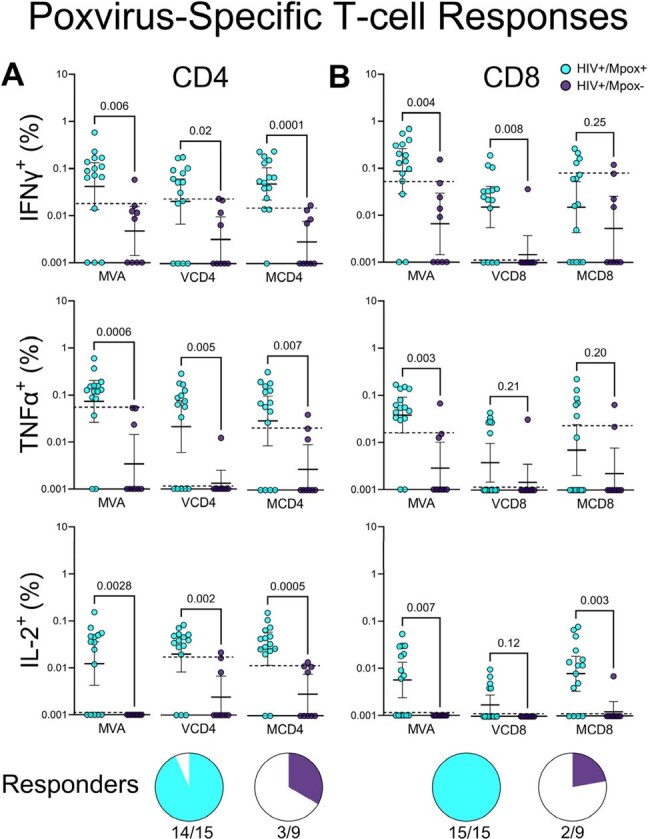

Poxvirus-specific T-cell responses in PLWH as determined by intracellular cytokine stimulation using live vaccinia virus (MVA), conserved orthopoxvirus peptide pools (VCD4 and VCD8), and mpox-specific peptide pools (MCD4 and MCD8). Pools provided by Alba Grifoni and Alessandro Sette (La Jolla Institute). Interferon-gamma (IFNγ), tumor necrosis factor alpha (TNFα), and interleukin 2 (IL-2) were the predominant cytokines detected. Responders defined as having any CD4 or CD8 response for any of the 9 stimulations above the 90th percentile of the control group.

**Results:**

Our assays differentiated well between the mpox-naïve and mpox-survivors. All mpox-survivors had binding titers of 10 to 1,000 times background with no antibodies detected in mpox-uninfected PLWH. Mpox-specific CD4 and CD8 responses of 0.01 to 0.3% were present in all infected individuals, comparable to immunocompetent mpox-survivors. The magnitude of mpox-specific T-cell responses correlated well with vaccinia-specific T-cell responses, demonstrating induction of broad anti-orthopoxvirus cell-mediated immunity. CD4 T-cell responses were T_H_1-type, with higher polyfunctionality in mpox-specific T-cells compared to HIV and CMV-specific T-cells. The magnitude of the IFNγ and TNFα CD4 and CD8 responses correlated with mpox-binding antibody titers, but did not predict cross-neutralization titers from vaccinia, a closely related virus that is used to vaccinate for smallpox and mpox. Host CD4 count did not correlate with the magnitude of mpox-specific cellular or humoral immunity.

**Conclusion:**

These new assays effectively quantify and characterize mpox-specific immunity, with strong induction of orthopoxvirus-specific humoral and cellular immunity in immunocompromised hosts with HIV. Although PLWH are at increased risk for complications from mpox, their potent immune responses post-infection suggest that most are at reduced risk for severe complications from re-infection.

**Disclosures:**

Samuel D. Stampfer, MD/PhD, Gilead: Stocks/Bonds (Public Company) Colleen F. Kelley, MD, MPH, Gilead: Grant/Research Support|Humanigen: Grant/Research Support|Moderna: Grant/Research Support|Novavax: Grant/Research Support|ViiV: Grant/Research Support

